# Unusual Fusion Proteins of HIV-1

**DOI:** 10.3389/fmicb.2016.02152

**Published:** 2017-01-09

**Authors:** Simon Langer, Daniel Sauter

**Affiliations:** Institute of Molecular Virology, Ulm University Medical CenterUlm, Germany

**Keywords:** HIV-1, fusion protein, gene fusion, alternative splicing, polymorphism, ribosomal frameshift

## Abstract

Despite its small genome size, the Human Immunodeficiency Virus 1 (HIV-1) is one of the most successful pathogens and has infected more than 70 million people worldwide within the last decades. In total, HIV-1 expresses 16 canonical proteins from only nine genes within its 10 kb genome. Expression of the structural genes *gag, pol*, and *env*, the regulatory genes *rev* and *tat* and the accessory genes *vpu, nef*, *vpr*, and *vif* enables assembly of the viral particle, regulates viral gene transcription, and equips the virus to evade or counteract host immune responses. In addition to the canonically expressed proteins, a growing number of publications describe the existence of non-canonical fusion proteins in HIV-1 infected cells. Most of them are encoded by the *tat*-*env*-*rev* locus. While the majority of these fusion proteins (e.g., TNV/p28^*tev*^, p18^6Drev^, Tat1-Rev2, Tat^8c, p17^tev^, or Ref) are the result of alternative splicing events, Tat-T/Vpt is produced upon programmed ribosomal frameshifting, and a Rev1-Vpu fusion protein is expressed due to a nucleotide polymorphism that is unique to certain HIV-1 clade A and C strains. A better understanding of the expression and activity of these non-canonical viral proteins will help to dissect their potential role in viral replication and reveal how HIV-1 optimized the coding potential of its genes. The goal of this review is to provide an overview of previously described HIV-1 fusion proteins and to summarize our current knowledge of their expression patterns and putative functions.

## Introduction

**Figure 1:**
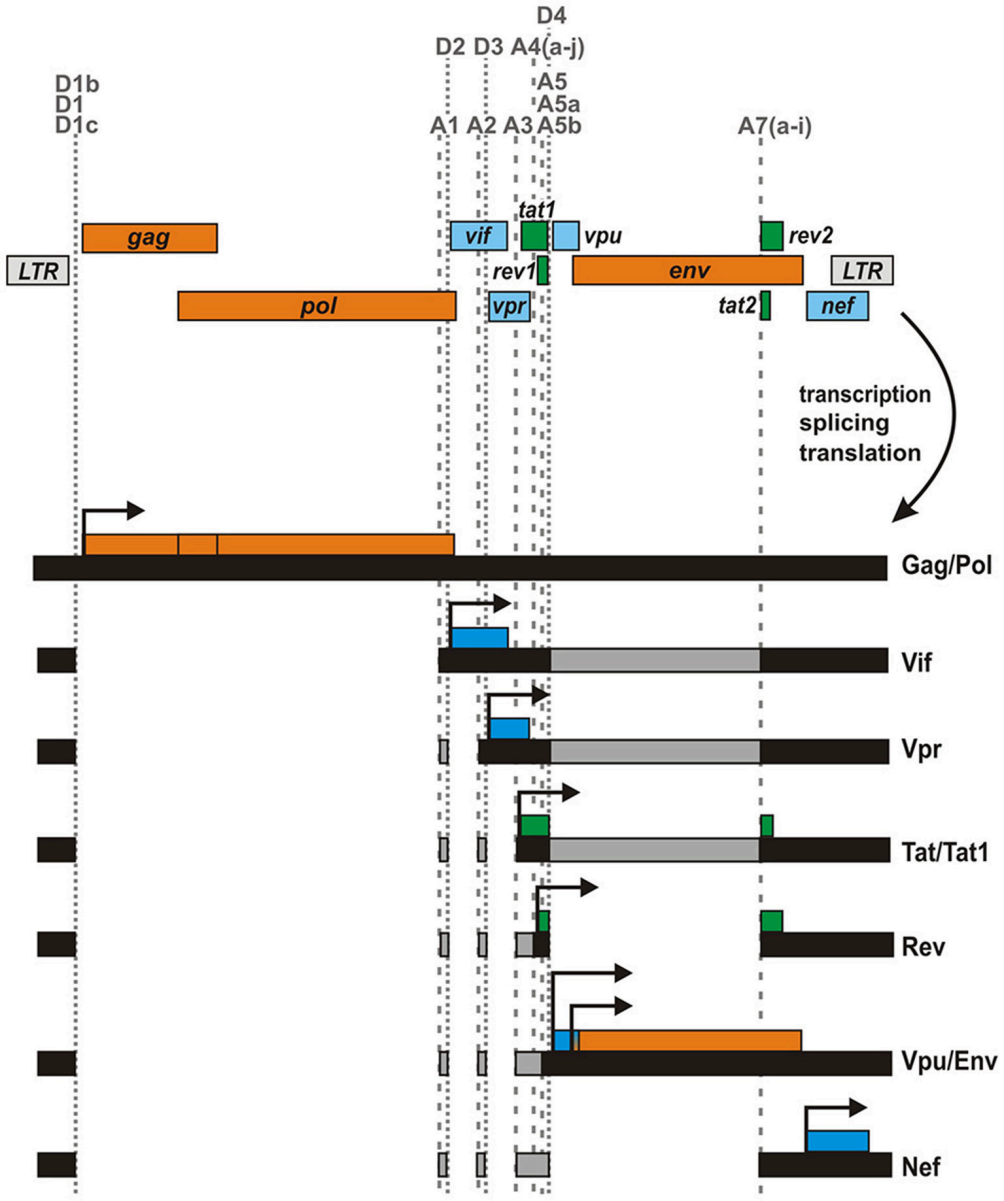
**Genome and major mRNA transcripts of HIV-1**. The HIV-1 genome comprises nine canonical genes that are arranged in three different reading frames. These genes encode for structural/enzymatic (orange), regulatory (green), and accessory (blue) proteins and are flanked by two long terminal repeats (LTR). The viral mRNA is spliced into more than 100 different mono- or multi-cistronic mRNA transcripts (Ocwieja et al., 2012), encoding different viral proteins. Splice donor (D) and acceptor (A) sites are indicted by dotted and dashed vertical lines, respectively.

The genome of the Human Immunodeficiency Virus type 1 (HIV-1), the major causative agent of the current AIDS pandemic, is relatively small, comprising <10,000 bases in total. Arranged in three different reading frames, it contains only nine canonical genes (Figure 1). Nevertheless, the virus replicates and spreads efficiently in its human host, which expresses about 2500 times more protein-coding genes from a three billion base pair genome. How does a retrovirus with its limited genome size manage to keep pace in this David vs. Goliath struggle? How can such a tiny genome encode all the tools that are required for efficient replication and immune evasion in such a hostile environment? One major advantage of HIV-1 and related retroviruses compared to their host species is certainly their high mutation rate that allows them to quickly adapt to an ever-changing environment. Furthermore, viral proteins are often multifunctional and exert a multitude of immune evasion activities. The paragon of such a multitasking or moonlighting protein is HIV-1 Nef, which has been described to downmodulate a variety of surface receptors including CD4, MHC class I, CD28, and CXCR4, counteracts the host restriction factors SERINC3/5, and upregulates the invariant chain/CD74 to suppress antigen presentation (Pereira and daSilva, 2016). Finally, viral genomes are often very compact, containing overlapping genes that encode for bi- and multi-cistronic mRNAs. As a result, viruses frequently utilize non-canonical translation mechanisms such as internal ribosomal entry, leaky scanning, ribosomal frameshifting, shunting, or reinitiation (Firth and Brierley, 2012). Another important mechanism increasing the coding capacity of viral genomes is alternative splicing. HIV-1 and related lentiviruses contain dozens of splice donor and acceptor sites that allow the generation of more than 100 different mRNA species (Ocwieja et al., 2012; Figure 1). Generation and translation of these mRNAs are tightly regulated throughout the viral replication cycle and enable the coordinated synthesis of structural, regulatory and accessory proteins in an optimized ratio. For example, expression of the HIV-1 regulatory proteins Tat and Rev requires the joining of two exons (*tat1/2* or *rev1/2*) via splicing at donor D4 and acceptor A7, whereas all four accessory proteins (Vif, Vpr, Vpu, and Nef) are encoded by mono-exonic genes. Notably, *vpu* overlaps with the viral envelope (*env*) gene and both are expressed from bicistronic mRNA species (Figure 1). Translation of downstream *env* is enabled by a weak Kozak sequence of *vpu* (leaky scanning) and/or ribosomal shunting mechanisms that allow to bypass upstream AUG codons (Anderson et al., 2007). While expression of Env as well as all accessory and regulatory proteins requires splicing, Gag and Pol are encoded by the 5′ half of the viral genome and expressed by unspliced viral mRNA. Gag can either be expressed alone or, upon ribosomal frameshifting, as a Gag-Pol poly-protein. The three precursor proteins Gag, Gag-Pol, and Env are proteolytically processed into mature proteins: Gag is cleaved by the viral protease into matrix, capsid, nucleocapsid, and the p6 protein. Similarly, the viral protease generates the mature viral enzymes reverse transcriptase (p51 and p66), protease and integrase from the Pol precursor protein. Finally, the Envelope protein is cleaved by the cellular protease furin into its mature subunits gp120 and gp41.

Considering the multitude of modulatory processes underlying the expression of viral proteins, it is not surprising that several studies have reported the expression of non-canonical fusion proteins by HIV-1. While the majority of these fusion proteins are the result of alternative splicing events joining regular or cryptic open reading frames, two of them are expressed only upon ribosomal frameshifting or gene rearrangements, respectively. The aim of this review article is to provide an overview of previously described fusion proteins of HIV-1. We will summarize our current knowledge on their expression and generation by different HIV-1 strains, discuss possible roles during the retroviral life cycle and critically review a potential relevance for viral replication.

## HIV-1 fusion proteins generated by alternative splicing

### TNV/p28*^*tev*^*

**Figure 2:**
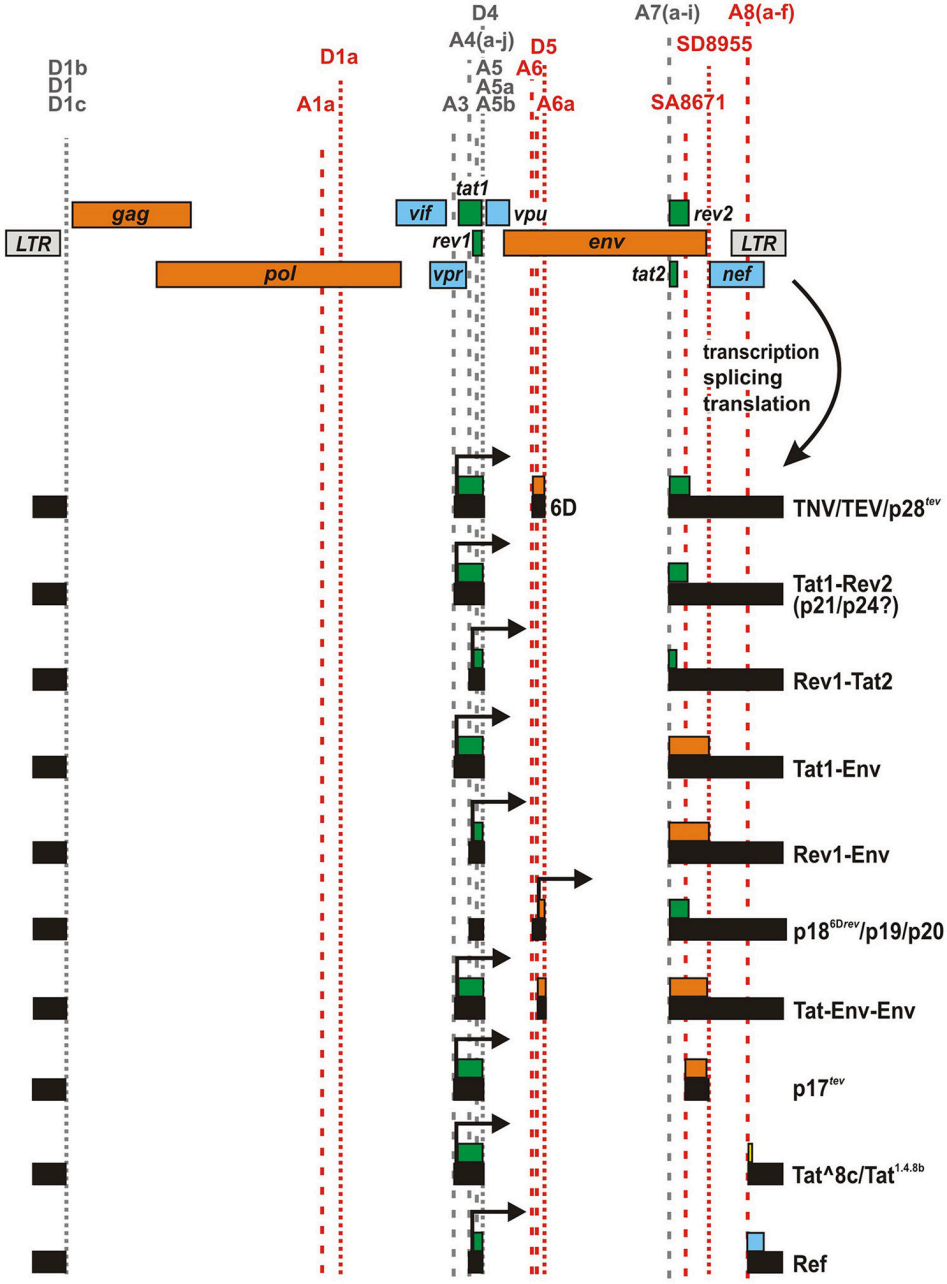
**HIV-1 fusion proteins generated by alternative splicing**. Several HIV-1 strains encode cryptic (red) and/or alternative splice sites. Splicing at these sites may result in the generation of mRNA species expressing unusual fusion proteins. For example, TNV, Tat1-Rev2, Tat1-Env, Rev1-Env, p18^6D*rev*^, Tat-Env-Env, p17^*tev*^, and Ref comprise (parts of) Tat1, Env, Rev2, and/or Nef that are fused together in frame. In case of Tat^8c, a few amino acids encoded by the *nef*/LTR overlap (that are not in frame with the *nef* ORF) are fused to the C-terminus of Tat1.

In addition to the canonical splice sites, several studies have reported the presence of alternative or cryptic splice sites in different clades of HIV-1 group M (Purcell and Martin, 1993; Ocwieja et al., 2012; Vega et al., 2016). While some of these sites are conserved among diverse HIV-1 isolates and seem to be used regularly, others have only been identified in single clones of HIV-1 and/or become only active upon mutation of canonical splice sites. Two well-described examples for cryptic sites are splice acceptor 6 (A6) and donor 5 (D5), which have been identified in the genomes of HXB2 and closely related subtype B strains (Figure 2). Utilization of these sites results in the generation of a small exon (116 bases) derived from the *env* open reading frame (ORF). cDNA analyses revealed that this exon (designated 6D) may be fused to *tat1* and *rev2* encoding exons via splice donor 4 (D4) and acceptor 7 (A7), respectively (Feinberg et al., 1986; Wright et al., 1986; Benko et al., 1990; Salfeld et al., 1990; Schwartz et al., 1990a).

Two groups demonstrated that the respective mRNA can be translated into an unusual tripartite fusion protein comprising Tat1, 38 amino acids of Env including its V1 loop, and Rev2 (Benko et al., 1990; Salfeld et al., 1990). Salfeld and colleagues found that this protein migrates at an apparent size of 26 kDa and was probably identical to the Tat-related protein p26 described in earlier studies (Feinberg et al., 1986; Wright et al., 1986). In reference to its parental proteins Tat, Env, and Rev, the fusion protein was named TNV (Salfeld et al., 1990). The group of Barbara Felber identified the same protein independently and termed it p28^*tev*^ as it migrated slightly slower in their experiments (Benko et al., 1990). Both studies analyzed the closely related HIV-1 M clade B HXB2 and/or HXB3 clones. While Salfeld et al. analyzed the expression of the fusion protein only in transfected COS-7 cells, Benko and colleagues demonstrated that TNV/p28^*tev*^ is also expressed in various human T cell lines infected with HIV-1 HXB2 (Table 1). In agreement with the finding that the N-terminus of Tat, encoded by *tat1*, is sufficient to transactivate viral transcription (Sodroski et al., 1985; Cullen, 1990; Vives et al., 1994), TNV/p28^*tev*^ also enhances LTR-mediated gene expression and may thus represent a *bona fide* regulatory protein. Reporter assays revealed that the transactivating activity of TNV/p28^*tev*^ is only about 30% lower than that of its parental Tat protein (Benko et al., 1990; Salfeld et al., 1990). In contrast, the fusion protein exerts no (Salfeld et al., 1990) or only weak (Benko et al., 1990) Rev activity. Due to its chimeric structure, TNV/p28^*tev*^ possesses two stretches of basic amino acids in its Tat1 and Rev2 domains that mediate nucleolar localization (Benko et al., 1990). The nuclear localization and absence of a signal peptide probably also prevents glycosylation despite the presence of four N-linked glycosylation sites in the Env-derived fragment (Salfeld et al., 1990). Radiolabeling revealed that TNV/p28^*tev*^ is weakly phosphorylated, probably at two phosphate acceptors near its C-terminus (Benko et al., 1990). This finding is in agreement with the observation that the fusion protein migrates as a doublet of closely spaced bands in SDS gels (Göttlinger et al., 1992).

**Table 1:** **HIV-1 fusion proteins**.

**Fusion protein**	**Parental proteins**	**Size**	**Function**	**Detected in/by**			**References**
				**Virus**	**Cell type**	**Method**	
TEV/p28*^tev^*	Tat1, Env, Rev2	28 kDa	Tat activity, weak Rev activity	HIV-1 M HXB2 and HXB3	Chronically infected MOLT-4/IIIB, H9/HXB2, H9/HXB3, TH4-7-5 and LC-5/HIVIIIB cells, transfected HeLa cells	IP, IF, WB, SB, sequencing	Benko et al., 1990; Schwartz et al., 1990a; Neumann et al., 1994
TNV	Tat1, Env, Rev2	26 kDa	Tat activity, no Rev activity, dispensable for replication	(Fragments of) HIV-1 M HXBc2	Transfected COS-1 and -7 cells, transfected murine CB2MX3-2 cells, reticulocyte extract	IP, cDNA phage library, IVT	Feinberg et al., 1986; Wright et al., 1986; Salfeld et al., 1990; Göttlinger et al., 1992
Tat/Rev chimeras (p21/p24?)	Tat, Rev	21, 24 kDa	Not analyzed	HIV-1 M NL4-3, HIV-1 M HXBc2 Primary HIV-1 M clade C (SPX10)	CD4+ CD25+ patient cells, infected PBMCs and HOS-CD4-CCR5 cells, transfected COS-7, and HeLa cells	IP, sequencing	Salfeld et al., 1990; Purcell and Martin, 1993; Ocwieja et al., 2012; Vega et al., 2016
Tat1-Env	Tat1, Env	~210 aa	Not analyzed	Primary HIV-1 M clade C (SPX10)	CD4+ CD25+ patient cells	Sequencing	Vega et al., 2016
Rev1-Env	Rev1, Env	159 aa	Not analyzed	HIV-1 M 89.6	Infected primary CD4+ T cells, infected HOS-CD4-CCR5 cells	Sequencing	Ocwieja et al., 2012
p18^6D*rev*^	Env, Rev2	18 kDa	No Rev activity	HIV-1 M HXB2 and pm213 L1	Chronically infected H9/HXB2, TH4-7-5 and CEM/pm213 cells, transfected HeLa cells	Sequencing, IP, IF, SB, WB,	Benko et al., 1990; Schwartz et al., 1990a; Neumann et al., 1994
p20	Env, Rev2	20 kDa	Not analyzed	HIV-1 M HXBc2	Reticulocyte extract	IVT, IP	Salfeld et al., 1990
p19	Env, Rev2	19 kDa	Not analyzed	HIV-1 M HXBc2	Transfected COS-7 cells	IP	Göttlinger et al., 1992
Tat-Env-Env	Tat1, Env	235 aa	Not analyzed	HIV-1 M 89.6	Infected primary CD4+ T cells, infected HOS-CD4-CCR5 cells	Sequencing	Ocwieja et al., 2012
p17^tev^	Tat1, Env	17 kDa	Weak Tat activity	HIV-1 M IIIB	Transfected COS cells, Reticulocyte extract	SB, IP, IVT, sequencing	Furtado et al., 1991
Tat^1.4.8b^	Tat1, Nef^shift^	90 aa	Not analyzed	Primary HIV-1 M clade B	PBMCs from an HIV-1 infected individual	Sequencing	Carrera et al., 2010
Tat^8c	Tat1, Nef^shift^	97 aa	Weak Tat activity	HIV-1 M 89.6	Infected primary CD4+ T cells, infected HOS-CD4-CCR5 cells	Sequencing	Ocwieja et al., 2012
Ref	Rev1, Nef	12.5 kDa	No Rev activity	HIV-1 M 89.6	Infected primary CD4+ T cells, infected HOS-CD4-CCR5 cells	WB, sequencing	Ocwieja et al., 2012
Tat-T/Vpt	Tat1, T (Tat1^shift^, Vpu^shift^)	17 kDa	No detectable Rev or Tat activity	(Fragments of) HIV-1 M HXBc2, BH10 and BRU	Reticulocyte extract	IVT, IP	Cohen et al., 1990
Rev1-Vpu	Rev1, Vpu	~14 kDa	Dispensable for replication	HIV-1 M clade A, C and CRF	Transfected HEK293T cells, infected SupT1 cells, and PBMCs	WB, sequencing	Kraus et al., 2010; Langer et al., 2015

IP, immunoprecipitation; IF, immunofluorescence; WB, Western blot; SB, Southern blot; IVT, in vitro translation; identical/similar proteins are separated by dashed lines.

To investigate the importance of TNV/p28^*tev*^ for viral replication, Göttlinger and colleagues mutated the A6 and D5 splice sites in *env* without altering its primary amino acid sequences. Experiments in Jurkat T cells and PBMCs revealed that the A6 mutant of HIV-1 HXBc2 replicated as efficiently as the respective wild type control (Göttlinger et al., 1992). These findings demonstrate that expression of TNV/p28^*tev*^ has no significant effect on viral replication, at least *in vitro*. Interestingly, the D5 mutant was replication-defective. However, this phenotype could be ascribed to the utilization of another cryptic splice donor that resulted in detrimental intron removal and possibly reduced Tat and Rev expression levels (Göttlinger et al., 1992). These results are in agreement with the observation that most HIV-1 strains lack the cryptic splice sites generating exon 6D (Göttlinger et al., 1992). In fact, mutations that increase the amount of TNV/p28^*tev*^ encoding mRNAs may be detrimental for viral replication as the expression of functional Rev is reduced (Göttlinger et al., 1992; Wentz et al., 1997).

### Tat1-Rev2 (p21, p24) and Rev1-Tat2 chimeras

Besides the TNV/p28^*tev*^ fusion protein, Salfeld and colleagues observed the expression of two additional proteins (p21, p24) that are detected by both Rev- and Tat-specific antisera (Salfeld et al., 1990). They hypothesized that at least one of these two proteins may represent an alternative Tat-Rev fusion product that is expressed if *tat1* is fused in frame to *rev2*, without any additional *env* sequences. Notably, comprehensive analyses of mRNA species in HIV-1 infected cells identified several neighboring splice acceptor sites at the 5′ end of *rev2/tat2* that introduce a frameshift and may result in the expression of various chimeric Rev1-Tat2 or Tat1-Rev2 proteins (Figure 2, Table 1) (Schwartz et al., 1990a; Purcell and Martin, 1993; Ocwieja et al., 2012; Vega et al., 2016). Although at least some of these splice sites can be found in diverse subtypes of (primary) HIV-1 group M isolates (Vega et al., 2016), the expression of chimeric Tat/Rev proteins and their possible role in viral replication has never been investigated.

### Tat1-Env and Rev1-Env chimeras

Alternative splicing at the Rev1-Rev2/Tat1-Tat2 junction may not only result in the production of Tat-Rev chimeras, but also entail the expression of unusual Rev-Env or Tat-Env fusion proteins (Figure 2, Table 1). For example, usage of acceptors A7g and h in conjunction with donor D4 results in a +2 frameshift that enables the expression of a Tat1-Env protein (Vega et al., 2016). Conversely, splicing at A7e induces a +1 frameshift and the resulting RNA species have the potential to express a Rev1-Env fusion protein (Ocwieja et al., 2012). Due to preferential usage of acceptor A5, however, the majority of mRNA species using alternative splice sites of A7 may lack the *rev1* and *tat1* initiation codons and express Nef instead (Ocwieja et al., 2012; Vega et al., 2016).

### p18^6**D***rev*^/p19/p20

Depending on the specific splice acceptor used, the cryptic *env* exon 6D can be fused to different exons at its 5′ end. Notably, a TNV/p28^*tev*^ fusion protein can only be synthesized upon usage of splice acceptor 3 (A3) since alternative utilization of A4 and A5 results in a loss of the *tat1* initiation codon (Figure 2). In the latter case, two methionine residues in exon 6D may serve as alternative start codons and result in the expression of a 6D/Env-Rev2 fusion protein (Göttlinger et al., 1992; Neumann et al., 1994). Experiments in transfected HeLa and COS-7 cells as well as chronically infected H9 and CEM cells revealed that at least the HIV-1 M HXB2 clone and HIV-1 pm213 L1, a closely related strain, are able to express this fusion protein. According to its apparent size in the gel, this unusual viral protein has been termed p18^6D*rev*^ (Benko et al., 1990; Schwartz et al., 1990a; Wentz et al., 1997), p19 (Göttlinger et al., 1992), or p20 (Salfeld et al., 1990). In contrast to TNV/p28^*tev*^, which is exclusively localized in nucleoli, p18^6D*rev*^ can be found in both nucleoli and the cytoplasm (Benko et al., 1990). Thus, the Env domain seems to affect the otherwise nuclear localization of Rev2. Furthermore, p18^6D*rev*^ did not display any significant Rev activity (Benko et al., 1990). This is in agreement with the finding that both the N- and C-terminal parts of Rev are required for nuclear targeting and functional activity of this regulatory protein (Malim et al., 1989).

### Tat-Env-Env

Analyzing the HIV-1 clone 89.6, Ocwieja and colleagues identified another cryptic splice acceptor site (named A6a) that lies 52 bp downstream of A6 (Ocwieja et al., 2012). RNAs generated via splicing at this site are predicted to encode a tripartite Tat-Env-Env fusion protein comprising the N-terminus of Tat and two stretches (aa 145–169 and 716–853) of Env (Figure 2, Table 1). Similar to A6, however, acceptor A6a is not well conserved among different strains of HIV-1. This is also true for splice donor D5, which is required for the generation of both, TNV/p28^*tev*^ and Tat-Env-Env encoding RNA (Ocwieja et al., 2012).

### p17*^tev^*

In 1991, Furtado and colleagues identified a novel splice acceptor site (SA8671) in *env*, located 240 bases downstream of the canonical *tat*/*rev* splice acceptor A7 (Figure 2). As a result, the *tat1* encoding exon may be fused in frame to an exon encoding the C-terminal 58 amino acids of Env gp41 (Furtado et al., 1991). Experiments in transfected COS cells and rabbit reticulocyte extracts demonstrated that the respective mRNA indeed expressed a 17 kDa protein (named p17^*tev*^) that can be detected by both Tat- and gp41-specific antibodies. RNase protection experiments, however, showed that p17^*tev*^ encoding mRNA is only expressed at very low levels, which may explain why the authors failed to detect this fusion protein in infected H9 cells or primary lymphocytes. Reporter assays revealed that p17^*tev*^ exerts only weak transactivating activity although it comprises the whole N-terminus of Tat, encoded by *tat1*. Unlike other mutated Tat proteins (Pearson et al., 1990), p17^*tev*^ did not exert any dominant negative effect on wild type Tat (Furtado et al., 1991).

### Tat^8c/Tat^1.4.8b^

In contrast to splice acceptors A6 and SA8671, which have only been detected in few lab-adapted clones of HIV-1, several groups reported the presence of additional splice acceptor sites (A8a–e) in the *nef* genes of both laboratory-adapted and primary isolates of HIV-1 (Smith et al., 1992; Carrera et al., 2010; Ocwieja et al., 2012) (Figure 2). These sites result in the generation of a previously unappreciated class of 1 kb transcripts. Intriguingly, A8c may be used as frequently as acceptor A7, which is required for expression of regular Rev and Tat proteins (Ocwieja et al., 2012). Splice events joining donor D5 to acceptors A8a–e result in mRNA species that have the potential to encode Tat1-Nef fusion proteins. For example, Carrera and colleagues predicted the expression of a Tat^1.4.8b^ protein upon splicing of D4 to A8b. This fusion protein consists of the N-terminus of Tat and 18 amino acids encoded by the *nef* /LTR region (Carrera et al., 2010). Notably, however, the 18 C-terminal amino acids do not contain any functional motifs of Nef, as *tat*^*1.4.8b*^ and *nef* are not translated in the same reading frame. More recently, Ocwieja and colleagues identified mRNA species in infected primary CD4+ T cells, which resulted from splicing of D4 to A8c (Ocwieja et al., 2012). These mRNAs express a Tat^8c fusion protein consisting of Tat1 and 25 novel amino acids encoded by the *nef* /LTR locus. In transfected TZM-bl cells, this protein exerted only weak transactivating activity. Notably, analyses of PBMCs from HIV-1 infected individuals demonstrated that acceptors A8 may also be fused to donor D1, resulting in RNA species that have the potential to encode a truncated protein, consisting of the C-terminal 34 amino acids of Nef (Smith et al., 1992; Carrera et al., 2010). Although the initiation codon of this protein, named C-Nef-34, is conserved among most clades of HIV-1 group M (Carrera et al., 2010) and although the majority of HIV-1 and HIV-2 strains contain at least one A8 site (Ocwieja et al., 2012), the importance of C-Nef-34 and/or Tat^8c for viral replication has remained unclear.

### Ref

Tat^8c/Tat^1.4.8b^ is not the only fusion protein containing amino acid sequences encoded by the *nef* /LTR locus of HIV-1. cDNA sequence analyses of cells infected with HIV-1 M 89.6 revealed the expression of mRNA species, in which an *rev1* encoding exon is joined to an exon containing the 3′ part of the *nef* ORF (Figure 2) (Ocwieja et al., 2012). These transcripts are the result of splicing events involving acceptors A4a–c and A8c, and encode a fusion of Rev1 and the C-terminal 80 amino acids of Nef. In reference to its parental proteins Rev and Nef, this fusion protein was named Ref. Although the amount of Ref encoding transcripts exceeded 20% of all completely spliced 1 kb mRNA species, a fusion protein was hardly detectable. A 12.5 kDa protein representing Ref became only detectable in transfected HEK293T cells treated with the proteasome inhibitor MG132. These findings suggest that the fusion protein is very unstable and are in agreement with the observation that Ref neither exerts Rev activity nor interferes with regular Rev function or HIV-1 particle production (Ocwieja et al., 2012).

## Expression of a Tat-T fusion protein (Vpt) upon ribosomal frameshifting

**Figure 3:**
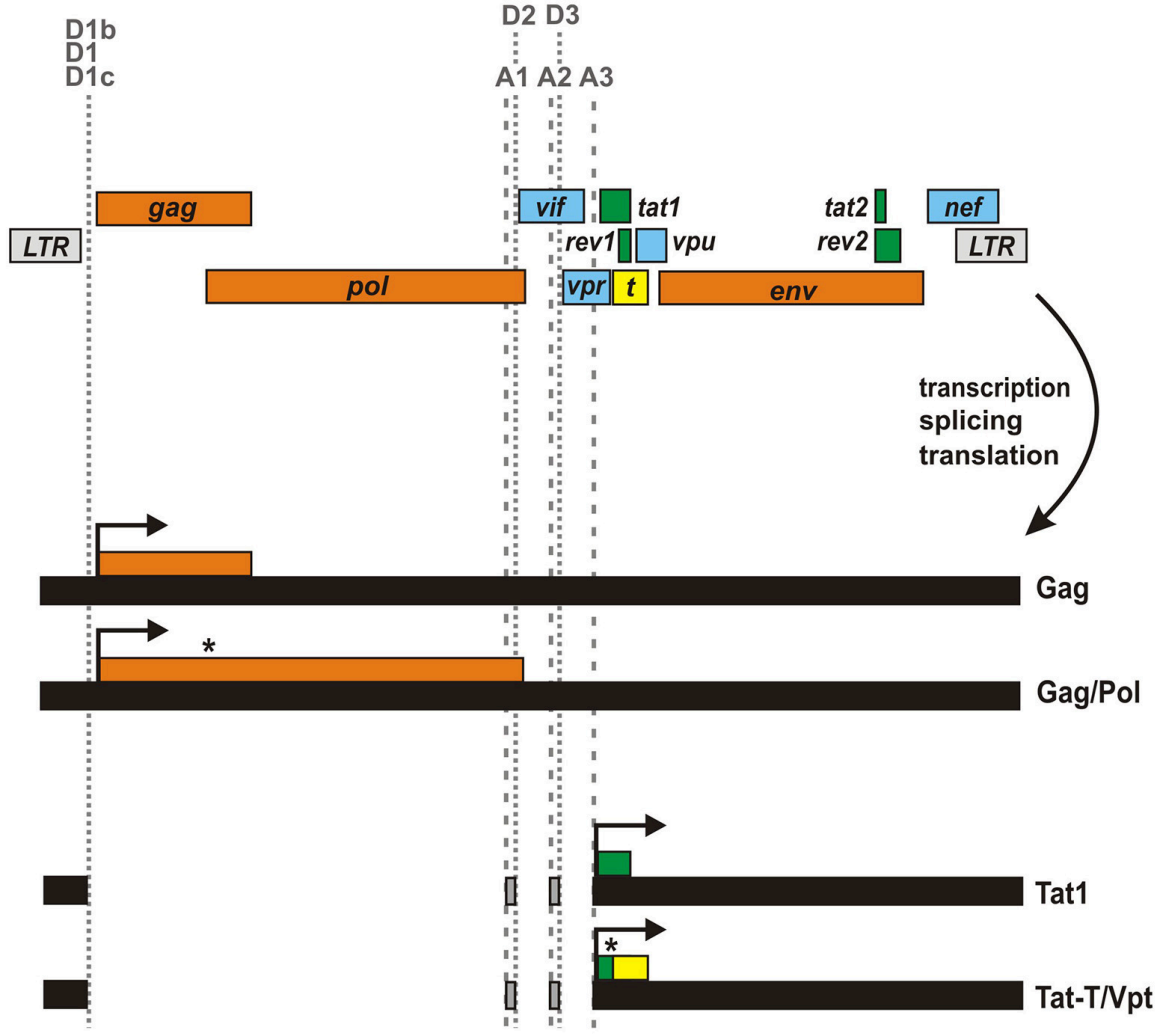
**Generation of Gag-Pol and Tat-T/Vpt by ribosomal frameshifting**. Unspliced viral mRNA contains a slippery sequence and a downstream RNA secondary structure in *gag* resulting in a frameshift event in about 5% of all translation events. This ribosomal frameshift signal (highlighted by an asterisk) enables the synthesis of the Gag/Pol polyprotein instead of Gag only. A similar frameshift signal in Tat1-encoding mRNA species has been suggested to result in the expression of an unusual protein, in which the N-terminus of Tat1 is fused to the T open reading frame (yellow) encoded by the *tat1/rev1/vpu* locus.

In addition to alternative splicing, ribosomal frameshifting represents another mechanism that may result in the expression of fusion proteins. The most prominent example in HIV-1 and related primate lentiviruses is the Gag/Pol polyprotein, which is the result of a −1 frameshift event in the *gag* ORF (Figure 3). The frameshift in *pol* depends on a stem-loop structure stalling the translocating ribosome and an upstream heptameric “slippery site” where ribosomal frameshifting occurs (Dinman et al., 2002). While the slippage heptamer (5′-UUUUUUA-3′) itself results in frameshifting, its frequency is increased to about 5% by the adjacent stem-loop structure (Kobayashi et al., 2010; Mouzakis et al., 2013). Interestingly, a similar combination of slippage sequence and RNA secondary structure can be found within the first exon of *tat* (Cohen et al., 1990). This second sequence (5′-UAAAAAG-3′) is highly conserved among HIV-1 strains (Steffy and Wong-Staal, 1991) and has been shown to result in the expression of a cryptic reading frame called T that overlaps with *rev1* and *vpu* (Figure 3) (Sonigo et al., 1985; Cohen et al., 1990). Due to a −1 frameshift, this T open reading frame (which does not harbor an initiation codon) is fused to the N-terminus of Tat1, resulting in the expression of a 17 kDa protein called Tat-T or Vpt. Although the frameshift signal is evolutionarily conserved, expression of this fusion protein in primary HIV-1 target cells is questionable. So far, this protein has only been detected upon *in vitro* translation using reticulocyte extract (Cohen et al., 1990). In fact, expression in infected T cells may be prevented by several splice sites disrupting the T open reading frame. In agreement with this, Tat-T/Vpt was not detectable in Jurkat and COS cells transfected with proviral HXBc2 DNA, and 50 different patient sera failed to detect expression of this fusion protein from an expression plasmid (Cohen et al., 1990). Finally, Tat-T does not exert any detectable Tat or Rev activity (Cohen et al., 1990). Nevertheless, even if Tat-T/Vpt is not expressed *in vivo*, it remains to be determined whether or how the frameshift sequence in *tat1* affects translation of regular Tat.

## Gene rearrangements enable the expression of a Rev1-Vpu fusion protein

**Figure 4:**
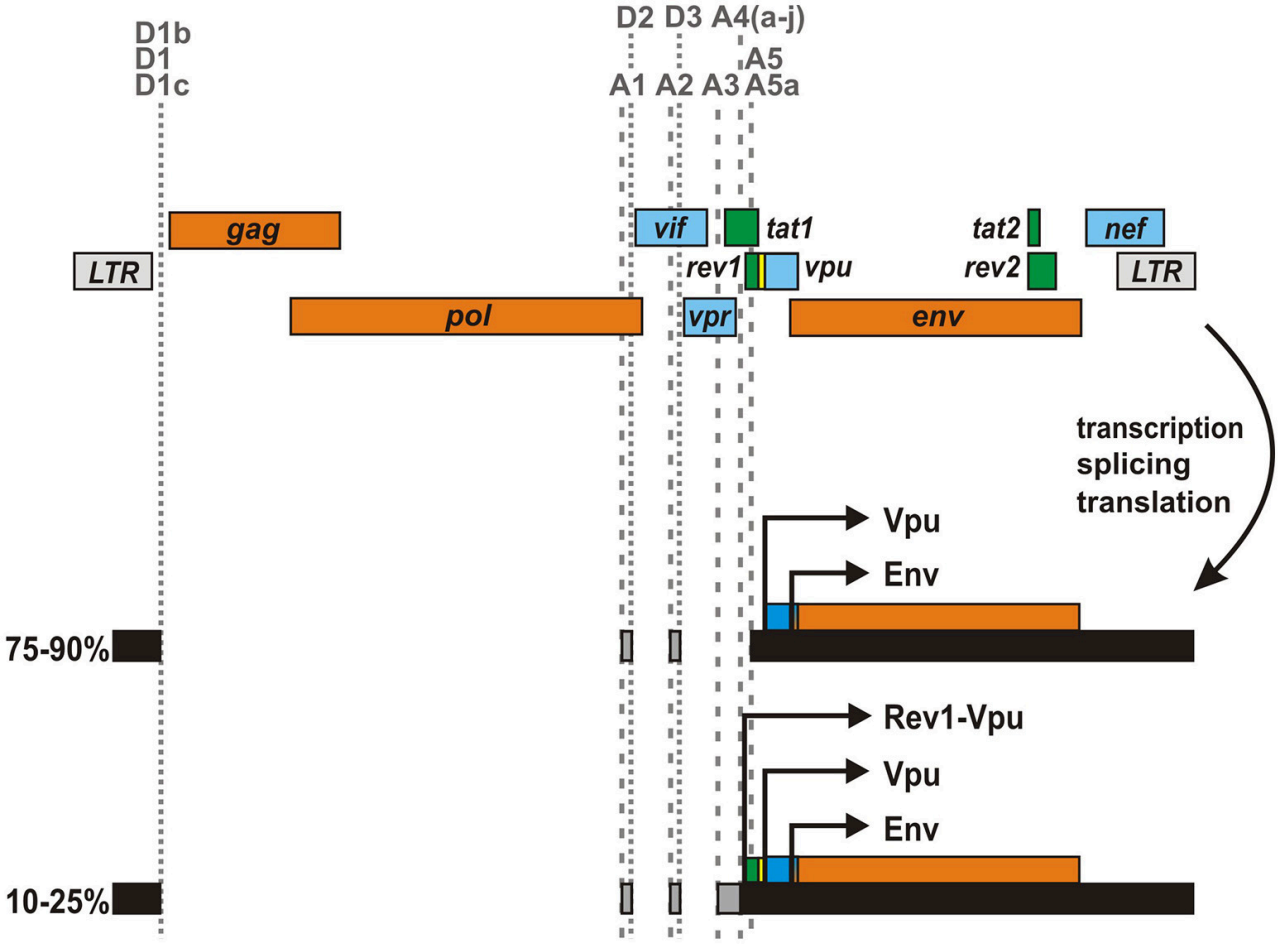
**Expression of a ***rev1-vpu*** fusion gene by certain HIV-1 strains**. In some HIV-1 M clade A and C strains, *rev1* and *vpu* are located in the same reading frame and not separated by an intervening stop codon. This unusual gene arrangement results in the expression of a Rev1-Vpu fusion protein from *vpu/env* encoding mRNA species. However, as the majority of *vpu/env* encoding mRNAs lack the *rev1* initiation codon, regular Vpu is expressed at much higher levels than Rev1-Vpu.

In the majority of HIV-1 strains, the *rev1* and *vpu* genes lie in different reading frames and/or are separated by an intervening stop codon (Figure 4). However, about 3% of clade A and 20% of clade C viruses as well as some circulating recombinants thereof encode an unusual *rev1-vpu* fusion gene (Kraus et al., 2010). Analysis of primary HIV-1 isolates harboring this ORF revealed that infected PBMCs express a Rev1-Vpu fusion protein of about 14 kDa (Langer et al., 2015). Although prevalence rates may be skewed by sampling biases, it is tempting to speculate that more than 10% of all circulating HIV-1 strains encode this unusual fusion protein, as subtype C viruses are responsible for about 50% of all infections worldwide (Osmanov et al., 2002; Hemelaar et al., 2006, 2011). Cells infected with *rev1-vpu* containing viruses, however, still express regular Vpu at much higher levels than Rev1-Vpu as most *vpu* encoding transcripts lack the initiation codon of *rev1* (Kraus et al., 2010; Ocwieja et al., 2012; Langer et al., 2015): in about 75-90% of all *vpu/env* mRNAs, an intron containing the start codon of Rev1 has been removed due to the usage of splice acceptor A5 (Purcell and Martin, 1993; Ocwieja et al., 2012). Only in 10–25% of the cases, A4 splice acceptors are used and the complete *rev1* ORF is retained. The expression of Rev1-Vpu may be further lowered by leaky scanning, in which the Rev1 initiation codon is skipped due to a weak Kozak sequence.

The characterization of virus pairs differing solely in their ability to express Rev1-Vpu revealed that the presence of this unusual fusion gene does not affect Rev-dependent nuclear export of incompletely spliced viral mRNAs. In agreement with the low Rev1-Vpu:Vpu ratio, downmodulation of CD4, tetherin counteraction and inhibition of NF-κB activation by Vpu were not affected either (Langer et al., 2015). Since the presence of *rev1-vpu* did not enhance viral replication in PBMCs or *ex vivo* infected tonsillar tissue, this gene arrangement does not seem to confer a selection advantage to HIV-1 *per*-*se*. Interestingly, however, mutations in the *rev1-vpu* intervening region strongly affected Env expression in some viruses (Langer et al., 2015), and the presence of the fusion gene in *rev/vpu/env* expression cassettes impeded pseudotyping of *env*-deficient viruses (Kraus et al., 2010). Previous studies demonstrated that HIV-1 optimizes Env expression throughout the course of infection to increase viral infectivity and transmission while minimizing antibody neutralization and immune activation (Parrish et al., 2013; Krapp et al., 2016). Thus, the expression of Rev1-Vpu may be merely an epiphenomenon of adaptive changes modulating Env expression. This hypothesis is in agreement with the description of several regulatory elements in the *rev1/vpu* region that may modulate leaky scanning and/or induce ribosomal shunting (Schwartz et al., 1990b; Anderson et al., 2007; Krummheuer et al., 2007). Together with the observation that the proportion of *rev1-vpu* encoding viruses does not seem to increase over time (unpublished data), these findings strongly suggest that the fusion gene itself has a neutral phenotype.

## Summary and concluding remarks

The generation of fusion proteins represents a mechanism of increasing the coding potential of viral genomes and has been identified in diverse viruses including primate lentiviruses, foamy, and papilloma viruses (Lambert et al., 1989; Viglianti et al., 1990; Lindemann and Rethwilm, 1998). By literally piecing together functional domains of different proteins, viruses may generate fusion products that retain or regulate the activity of their parental proteins and/or even exert entirely novel functions. Although HIV-1 is among the best characterized viruses, relatively little is known about its “fuseome,” i.e., the entity of all viral fusion genes and proteins. To date, more than a dozen non-canonical lentiviral fusion proteins have been described (Table 1). While two of them, Tat-T/Vpt and Rev1-Vpu, are the result of ribosomal frameshifting and genetic rearrangements, respectively, the remaining ones are expressed due to alternative splicing events. Although for some of them, expression has been confirmed on both mRNA and protein levels, a relevant role for all of these fusion proteins in lentiviral replication remains questionable for several reasons: (1) Most of the fusion proteins were only identified in a few laboratory-adapted viruses. For example, cryptic exon 6D, which is required for the generation of TNV/p28^*tev*^ and p18^6D*rev*^, has only been described for HIV-1 HXB2 and a few closely related viruses (Feinberg et al., 1986; Wright et al., 1986; Benko et al., 1990; Salfeld et al., 1990; Schwartz et al., 1990a; Göttlinger et al., 1992; Neumann et al., 1994; Wentz et al., 1997). Follow-up studies including *in vivo* transcriptome analyses of patient-derived cells failed to detect 6D transcripts in other HIV-1 strains and subtypes suggesting that they might represent an artifact of laboratory-adapted viruses (Furtado et al., 1991; Smith et al., 1992; Purcell and Martin, 1993; Vega et al., 2016). To our knowledge, Rev1-Vpu is the only unusual fusion protein known to be expressed by intact primary isolates of HIV-1 (Langer et al., 2015). (2) The total cellular levels of many fusion proteins are very low. P17^*tev*^ and Tat-T/Vpt, for example, were detectable upon *in vitro* translation, but not in transfected or infected T cells (Cohen et al., 1990; Furtado et al., 1991). Similarly, Ref was not detectable by Western blotting unless the cells were treated with a proteasome inhibitor (Ocwieja et al., 2012). (3) Although some fusion proteins were shown to exert the activity of their parental proteins, several mutational analyses argue against a crucial role of known fusion proteins in viral replication. Mutation of the splice sites generating exon 6D, for example, revealed that TNV/p28^*tev*^ is not required for efficient replication of HIV-1 in CD4+ T cells (Göttlinger et al., 1992). In fact, elevated usage of exon 6D may even be detrimental for viral replication (Wentz et al., 1997). Similarly, the majority of primary HIV-1 isolates seems to do well without a *rev1-vpu* fusion gene, and gain-of-function mutations did not enhance viral replication in PBMCs or lymphoid tissue (Kraus et al., 2010; Langer et al., 2015).

The observation that fusion proteins are expressed only by a fraction of HIV-1 strains and may be dispensable for viral replication *in vivo* strongly suggests that their expression is just a tolerated epiphenomenon of other adaptive changes. In line with this hypothesis, several studies suggested that cryptic splice sites such as A6 may have evolved to stabilize adjacent suboptimal splice sites and/or increase mRNA stability to balance the ratio of spliced and unspliced HIV-1 transcripts (Lu et al., 1990; Haseltine and Wong-Staal, 1991; Göttlinger et al., 1992; Lützelberger et al., 2006). Furthermore, novel splice sites may also be an (inevitable) result of adaptive changes in regulatory RNA elements, such as shunting structures or Kozak sequences. Mutations generating a *rev1-vpu* fusion gene, for example, have been shown to drastically affect *env* expression (Langer et al., 2015). Finally, fusion proteins may evolve to compensate for detrimental mutations elsewhere in the genome. One striking example has been described by the Berkhout lab, where a Tat-Rev fusion protein evolved to compensate for a mutation of the *rev* initiation codon (Verhoef et al., 2001). The observed Tat-Rev fusion comprised all domains of Rev and allowed the virus to replicate almost as efficiently as the respective wild type control.

No matter whether HIV-1 fusion proteins represent beneficial helpers, neutral factors or even detrimental byproducts, all of them may potentially be immunogenic and serve as T cell epitopes and/or antibody binding sites. To better assess their relevance for viral replication and immune activation, it is therefore crucial to investigate viral mRNA and protein expression in a broad and unbiased manner. Since viral gene expression seems to depend on the cell type and the viral strain (Ocwieja et al., 2012; Vega et al., 2016) rather than the time point of infection (Saltarelli et al., 1996), it is especially important to perform analyses in primary target cells infected with diverse groups and clades of HIV-1. For example, the recent pyrosequencing of CD4+ CD25+ lymphocytes from individuals infected with non-B subtypes revealed that the diversity of splice site usage and the expression of non-canonical transcripts is substantially higher than previously anticipated (Vega et al., 2016). Furthermore, Ocwieja and colleagues hypothesized that cryptic splice donor sites near the 3′ end of the viral RNA such as SD8955 or D6 may also be joined with adjacent exons of the host and result in the expression of chimeric viral-host proteins as previously described for self-inactivating (SIN) retroviral vectors (Almarza et al., 2011; Ocwieja et al., 2012). Remarkably, even defective proviruses that fail to produce infectious viral particles have recently been shown to express RNA species with unusual exon combinations (Imamichi et al., 2016). Due to large (intron) deletions, these unspliced RNAs may be exported from the nucleus in a Rev/RRE-independent manner, where they are predicted to produce truncated and/or chimeric viral proteins.

Thus, it is very likely that the lentiviral fuseome will further increase, and future analyses will reveal whether some HIV-1 strains express non-canonical fusion proteins with relevant functions *in vivo*.

## Author contributions

SL and DS wrote this review article.

### Conflict of interest statement

The authors declare that the research was conducted in the absence of any commercial or financial relationships that could be construed as a potential conflict of interest.
